# Relationship between nailfold capillaroscopy parameters and the severity of diabetic retinopathy

**DOI:** 10.1007/s00417-023-06220-z

**Published:** 2023-10-24

**Authors:** Tatsu Okabe, Hiroshi Kunikata, Masayuki Yasuda, Shinjiro Kodama, Yuta Maeda, Joe Nakano, Dan Takeno, Nobuo Fuse, Toru Nakazawa

**Affiliations:** 1 Department of Ophthalmology, Tohoku University Graduate School of Medicine, 1-1 Seiryo, Aoba, Sendai, Miyagi 980-8574 Japan; 2 Department of Retinal Disease Control, Tohoku University Graduate School of Medicine, Sendai, Japan; 3 Department of Metabolism and Diabetes, Tohoku University Graduate School of Medicine, Sendai, Japan; 4 At Co., Ltd., Osaka, Japan; 5 Department of Integrative Genomics, Tohoku Medical Megabank Organization, Tohoku University, Sendai, Japan; 6 Department of Advanced Ophthalmic Medicine, Tohoku University Graduate School of Medicine, Sendai, Japan; 7 Department of Ophthalmic Imaging and Information Analytics, Tohoku University Graduate School of Medicine, Sendai, Japan

**Keywords:** Nailfold capillaries, Nailfold capillaroscopy, Diabetic retinopathy, Biomarker

## Abstract

**Purpose:**

To determine whether non-invasive measurements of the nailfold capillaries (NCs) are associated with the presence and severity of diabetic retinopathy (DR) in patients with type 2 diabetes.

**Methods:**

Eighty-three eyes of 83 patients with type 2 diabetes were enrolled. Sixty-three age-matched non-diabetic subjects served as controls. Diabetic patients were classified by the severity of their DR: non-DR (NDR), non-proliferative DR (NPDR), and proliferative DR (PDR). We used nailfold capillaroscopy to measure NC parameters, including number, length, width, and turbidity.

**Results:**

Four NC parameters in the diabetic patients were significantly lower than in the controls (all *P* < 0.001). There was a statistically significant decrease in the NC parameters along with the increasing severity of DR (number: *P* = 0.02; all others: *P* < 0.001). Logistic regression analysis revealed that combining the systemic characteristics of age, sex, systolic blood pressure, estimated glomerular filtration rate, hemoglobin A1c level, and history of hypertension and dyslipidemia could indicate the presence of DR and PDR (the area under the receiver operating characteristic curve [AUC] = 0.81, *P* = 0.006; AUC = 0.87, *P* = 0.001, respectively). Furthermore, the discriminative power of DR was significantly improved (*P* = 0.03) by adding NC length to the systemic findings (AUC = 0.89, *P* < 0.001).

**Conclusion:**

NC measurement is a simple and non-invasive way to assess the risk of DR and its severity.


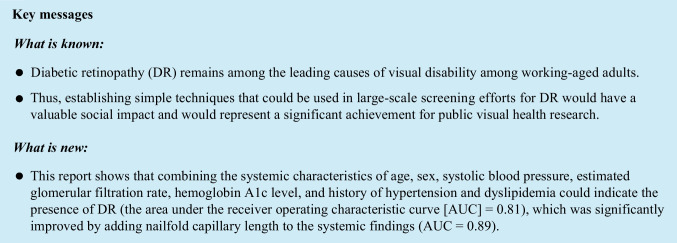


## Introduction

Diabetes mellitus (DM) is a serious medical and social problem impacting an estimated 366 million individuals globally. It is characterized by chronic hyperglycemia and pathological changes to the microvasculature. Blindness caused by this chronic condition is known as diabetic retinopathy (DR). A third of all diabetic patients develop DR and have a high mortality rate due to cardiovascular and renal deficiency. DR remains among the leading causes of visual disability in working-aged adults. Diabetic macular edema (DME) and proliferative diabetic retinopathy (PDR) carry particularly poor prognoses and can result in permanent visual impairment, despite the availability of treatment options such as medical therapy, laser photocoagulation, and vitreous surgery. This is due in part to the lack of a specific biomarker of DR that can easily be used without a specialized ophthalmologic examination. This situation has led to an increase in the number of patients with untreated, longstanding DR. Thus, establishing simple techniques that could be used in large-scale screening efforts for DR would have a valuable social impact and would represent a significant achievement for public visual health research.

The nailfold capillaries (NCs) are a component of the capillary microvasculature that can serve as indicators of vascular damage caused by various diseases, including diabetes, sleep disorders, Alzheimer’s dementia, stroke, and autoimmune connective tissue diseases [1–6]. Nailfold capillaroscopy, a non-invasive and straightforward tool for evaluating microvascular structure, has been used to assess cutaneous capillary damage in connective tissue diseases. More recently, the relationship of NC parameters with DM and DR has been explored. Some studies suggest an association between a higher number of NC crossings and DR, while others point to increased NC width and branching, as well as alterations such as increased tortuosity, bushy NCs, aneurysm, neoformation, and bizarre NCs as possible indicators of DR [7–9]. Nonetheless, previous reports have not used automatic assessment of NC parameters, making it difficult to eliminate the effects of grader subjectivity. Therefore, specific NC structural alterations have not generally been examined in diabetic patients, and thus the association of NC alterations with DR remains a subject of debate.

Recently, we developed a real-time microscope device (Kekkan-Bijin SC-10, At Co., Ltd., Osaka, Japan) to observe the NC vessels, and we succeeded in developing a method for the automatic quantification of NC images within a few seconds. In the present study, we assessed structural abnormalities of the NCs and their relationship with the presence and severity of DR using this newly developed NC analysis system. The objective of this report, therefore, is to determine whether quantitatively measured parameters of the NCs are associated with the presence and severity of DR in patients with type 2 diabetes.

## Methods

### Study design and subjects

This study evaluated outpatients between the ages of 35-70 years between January 2020 and November 2022 at the Sendai Open Hospital (Sendai, Japan), the Tohoku Medical Megabank Organization (Sendai, Japan), and the Department of Ophthalmology of Tohoku University Hospital (Sendai, Japan). A total of 83 outpatients with type 2 DM and 63 subjects without diabetes of similar sex and age were enrolled. Patients with type 1 diabetes; gestational diabetes; rheumatic disease; and retinal diseases other than DR, such as age-related macular degeneration, central serous chorioretinopathy, retinal vein occlusion, or retinal artery occlusion or ocular media opacity impeding clear fundus visualization, were excluded. The present cross-sectional study adhered to the principles outlined in the Declaration of Helsinki, and the protocols were granted approval by the clinical research ethics committee of the Tohoku University Graduate School of Medicine (protocol number 2022‐1-353, 2021‐1-1000‐1, 2019‐1-037). Informed consent was obtained from all participants involved in this study.

### Nailfold capillaroscopy

We employed the capillary microscope (Kekkan-Bijin SC-10; field of observation 500 μm × 700 μm) outfitted with the Capillary Analysis System program (CAS, At Co., Ltd.), which facilitates automatic quantification of NC images within the subungual space of the fourth finger on the left hand [10]. The fourth finger on the left hand was chosen as it possesses more transparent skin, is less susceptible to unexpected trauma, and is used less frequently in daily life than the other fingers [11].

NC measurements were conducted according to the following protocol: (1) apply oil to the nailfold of the left finger in order to create a smooth surface and reduce light reflection, (2) position the finger on the designated stage of the capillary scope, (3) adjust the position of the finger and manually focus on the NCs, (4) capture 5 images in 3 locations and save the captured images on a PC. NC measurements were performed throughout the year in all seasons at a room temperature of approximately 20° C.

The obtained NC images were quantitatively evaluated for number, length, width, and turbidity. The number of NCs was counted as the number of vessel parietals above the horizontal line in the center of each image. The length and width of the NCs were averaged after measuring the vertical and horizontal dimensions of the blood vessels, respectively. Turbidity of the NCs, which indicates the degree of cloudiness of the interstitial fluid between cells and blood vessels, was calculated using a proprietary method in the CAS. In the CAS, weaker turbidity is indicated by higher values and stronger turbidity by lower values.

### Measurement of clinical findings

Data were collected for medical history, the results of a clinical examination that included anthropometric measurements (weight, height, and body mass index [BMI]), systolic blood pressure (SBP), diastolic blood pressure (DBP), and the results of blood sample testing (performed according to automated standardized laboratory techniques) for total cholesterol, high-density lipoprotein (HDL), low-density lipoprotein (LDL), triglycerides (TG), hemoglobin A1c (HbA1c), and estimated glomerular filtration rate (eGFR). The presence and severity of DR were diagnosed through a baseline ophthalmological assessment of visual acuity and intraocular pressure, a slit-lamp examination, a dilated-pupil fundus examination, and fundus photography (DRI OCT Triton, Topcon, Tokyo, Japan). Only one eye from each patient was included in the data analysis; the clinically more severe eye was chosen in all cases. In cases where the severity of DR was similar in the right and left eyes, the right eye was included. The participants with diabetes were classified into three groups in accordance with the Early Treatment of Diabetic Retinopathy (ETDRS) standards: 30 patients had non-DR (NDR), 24 patients had non-proliferative DR (NPDR), and 29 patients had PDR.

### Statistical analysis

The statistical analysis was conducted using R software (version 4.2.2; R Foundation for Statistical Computing, Vienna, Austria). Descriptive statistics, including the mean and standard deviation (SD), were used for quantitative variables and frequency (n) and percentage (%) were used for qualitative variables. The Mann‐Whitney U test and the Kruskal-Wallis test were applied to assess differences between groups. The chi-square test was employed for frequency data on sex and history of hypertension (HT) and dyslipidemia (DL). A receiver operating characteristic (ROC) curve analysis was used to investigate the discriminative abilities of nailfold capillaroscopic alterations in DR and PDR. The Delong test was applied to examine the area under the ROC curve (AUC) of systemic factors alone and the AUC after adding NC length to the systemic factors. A multivariate logistic analysis was performed with the presence of DR or PDR as the objective variable and each NC parameter as the explanatory variable, with adjustment for age, sex, SBP, history of HT and DL, eGFR, and HbA1c. A *P*-value < 0.05 was considered statistically significant.

## Results

The clinical characteristics of both the controls and the patients with diabetes are shown in Table 1. The mean ± SD for age was 57.8 ± 7.5 years in the control group and 57.9 ± 8.6 years in the diabetes group, with no statistically significant difference between the two groups (*P* = 0.65). The male/female ratio was 44/19 in the control group and 50/33 in the diabetes group, with no statistically significant difference (*P* = 0.23). No statistically significant differences were observed in BMI or TG. However, all other measurements, including SBP, DBP, history of HT and DL, blood sample testing results, and NC parameters, were significantly different between the groups. HbA1c, SBP, DBP, and the ratio of HT and DL were statistically significantly higher in the diabetes group than in the control group (HbA1c: 7.45 ± 1.47% [58.0 ± 16.2 mmol/ml] versus 5.60 ± 0.32% [37.7 ± 3.5 mmol/ml], *P* < 0.001; SBP: 134.2 ± 19.8 mmHg versus 125.3 ± 15.5 mmHg, *P* = 0.009; DBP: 82.1 ± 12.1 mmHg versus 74.0 ± 12.1 mmHg, *P* < 0.001; HT: 71.1% versus 23.8%, *P* < 0.001; DL: 57.8% versus 11.1%, *P* < 0.001). Total cholesterol, HDL, LDL, and eGFR were significantly lower in the diabetes group than in the control group (total cholesterol: 195.9 ± 45.9 mg/dl versus 219.8 ± 35.5 mg/dl, *P* < 0.001; HDL: 53.1 ± 13.9 mg/dl versus 59.8 ± 16.3 mg/dl, *P* = 0.03; LDL: 111.9 ± 37.4 mg/dl versus 134.2 ± 28.8 mg/dl, *P* < 0.001; eGFR: 65.5 ± 24.8 ml/min/1.73 m^2^ versus 73.2 ± 12.2 ml/min/1.73 m^2^, *P* = 0.02). The proportion of glaucoma patients was significantly higher in the diabetes group than in the control group (7.2% versus 0%). All NC parameters were significantly lower in the diabetes group than in the control group (number: 4.24 ± 1.57 versus 5.52 ± 1.28, *P* < 0.001; length: 774 ± 569 versus 1445 ± 760, *P* < 0.001; width: 23.6 ± 8.4 versus 28.4 ± 7.9, *P* < 0.001; turbidity: 0.354 ± 0.162 versus 0.471 ± 0.164, *P* < 0.001). The characteristics of each DR severity group are shown in Table 2. There were significant differences in SBP and eGFR (SBP was 127.0 ± 13.6 mmHg in the NDR group, 136.8 ± 24.2 mmHg in the NPDR group, and 139.4 ± 19.5 mmHg in the PDR group [*P* = 0.02]; eGFR was 75.6 ± 22.7 ml/min/1.73 m^2^ in the NDR group, 63.6 ± 23.5 ml/min/1.73 m^2^ in the NPDR group, and 56.2 ± 24.9 ml/min/1.73 m^2^ in the PDR group [*P* = 0.004]). There were no significant differences in age, sex, BMI, DBP, history of HT and DL, or other blood sample testing results. There were no significant differences in the proportion of glaucoma patients at each DR stage. There were significant differences in NC parameters: Number was 4.73 ± 1.36 in the NDR group, 4.33 ± 1.77 in the NPDR group, and 3.66 ± 1.44 in the PDR group (*P* = 0.02). Length was 1183 ± 556, 546 ± 479, and 538 ± 384 in the groups, respectively (*P* < 0.001). Width was 28.7 ± 8.4, 21.3 ± 7.6, and 20.3 ± 6.4 in the groups, respectively (*P* < 0.001). Turbidity was 0.449 ± 0.138, 0.291 ± 0.157, and 0.308 ± 0.141 in the groups, respectively (*P* < 0.001).

**Table 1:** Clinical characteristics of age-matched non-diabetic controls and diabetic patients

	Controls	Diabetes	*P* value
Number of subjects	63	83	
Age (years)	57.8 ± 7.5	57.9 ± 8.6	0.65
Sex (M/F)	44/19	50/33	0.23
HbA_1c_ (%)	5.60 ± 0.32	7.45 ± 1.47	< 0.001
HbA_1c_ (mmol/mol)	37.7 ± 3.5	58.0 ± 16.2	< 0.001
BMI (kg/m^2^)	24.9 ± 3.4	26.4 ± 4.6	0.05
SBP (mmHg)	125.3 ± 15.5	134.2 ± 19.8	0.009
DBP (mmHg)	74.0 ± 12.1	82.1 ± 12.1	< 0.001
HT (n, %)	15, 23.8	59, 71.1	< 0.001
DL (n, %)	7, 11.1	48, 57.8	< 0.001
Total cholesterol (mg/dl)	219.8 ± 35.5	195.9 ± 45.9	< 0.001
HDL (mg/dl)	59.8 ± 16.3	53.1 ± 13.9	0.03
LDL (mg/dl)	134.2 ± 28.8	111.9 ± 37.4	< 0.001
TG (mg/dl)	130.4 ± 63.4	155.2 ± 92.4	0.09
eGFR (ml/min/1.73 m^2^)	73.2 ± 12.2	65.5 ± 24.8	0.02
Glaucoma (n, %)	0, 0	6, 7.2	0.03
NC parameters			
Number	5.52 ± 1.28	4.24 ± 1.57	< 0.001
Length (µm)	1445 ± 760	774 ± 569	< 0.001
Width (µm)	28.4 ± 7.9	23.6 ± 8.4	< 0.001
Turbidity	0.471 ± 0.164	0.354 ± 0.162	< 0.001

*HbA*_*1c*_, hemoglobin A1c; *BMI*, body mass index; *SBP*, systolic blood pressure; *DBP*, diastolic blood pressure; *HT*, Hypertension; *DL*, Dyslipidemia; *HDL*, high-density lipoprotein; *LDL*, low-density lipoprotein; *TG*, triglyceride; *eGFR*, estimated glomerular filtration rate; *NC,* nailfold capillary. Differences between groups were analyzed with the Mann-Whitney U test. The Chi-square test was used for frequency data on sex, HT, DL, and glaucoma. Differences were considered significant at the *P* < 0.05 level

**Table 2:** Clinical characteristics in each group of patients with diabetes

Severity of DR	NDR	NPDR	PDR	*P* value
Number of patients	30	24	29	
Age (years)	58.0 ± 9.4	59.7 ± 7.3	56.4 ± 9.0	0.46
Sex (M/F)	20/10	14/10	16/13	0.64
HbA_1c_ (%)	7.04 ± 0.98	7.65 ± 1.47	7.73 ± 1.84	0.28
HbA_1c_ (mmol/mol)	53.4 ± 10.6	60.1 ± 16.1	61.0 ± 20.1	0.28
BMI (kg/m^2^)	26.5 ± 4.0	26.2 ± 5.5	26.5 ± 4.4	0.77
SBP (mmHg)	127.0 ± 13.6	136.8 ± 24.2	139.4 ± 19.5	0.02
DBP (mmHg)	80.6 ± 12.0	82.0 ± 12.1	83.7 ± 12.5	0.64
HT (n, %)	18, 60.0	19, 79.2	22, 75.9	0.33
DL (n, %)	17, 56.7	17, 70.8	14, 48.3	0.25
Total cholesterol (mg/dl)	193.4 ± 28.9	187.4 ± 34.9	205.0 ± 64.6	0.74
HDL (mg/dl)	55.0 ± 15.9	51.9 ± 12.9	51.9 ± 12.3	0.58
LDL (mg/dl)	109.9 ± 26.2	102.0 ± 30.7	122.2 ± 49.9	0.42
TG (mg/dl)	165.9 ± 85.0	156.2 ± 112.8	143.0 ± 84.5	0.41
eGFR (ml/min/1.73 m^2^)	75.6 ± 22.7	63.6 ± 23.5	56.2 ± 24.9	0.004
Glaucoma (n, %)	1, 3.3	2, 8.3	3, 10.3	0.56
NC parameters				
Number	4.73 ± 1.36	4.33 ± 1.77	3.66 ± 1.44	0.02
Length (µm)	1183 ± 556	546 ± 479	538 ± 384	< 0.001
Width (µm)	28.7 ± 8.4	21.3 ± 7.6	20.3 ± 6.4	< 0.001
Turbidity	0.449 ± 0.138	0.291 ± 0.157	0.308 ± 0.141	< 0.001

*HbA*_*1c*_, hemoglobin A1c; *BMI*, body mass index; *SBP*, systolic blood pressure; *DBP*, diastolic blood pressure; *HT*, Hypertension; *DL*, Dyslipidemia; *HDL*, high-density lipoprotein; *LDL*, low-density lipoprotein; *TG*, triglyceride; *eGFR*, estimated glomerular filtration rate; *NC,* nailfold capillary; *DR*, diabetic retinopathy; *NDR*, non-diabetic retinopathy; *NPDR*, non-proliferative diabetic retinopathy; *PDR*, proliferative diabetic retinopathy. Differences between groups were tested with the Kruskal-Wallis test. The chi-square test was used for frequency data on sex, HT, DL, and glaucoma. Differences were considered significant at the *P* < 0.05 level

**Fig. 1:**
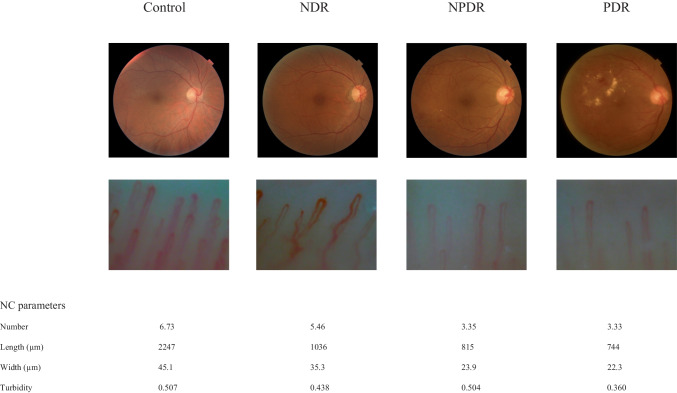
Fundus photographs and nailfold capillaroscopy images from representative cases, Left: control case. Middle left: NDR case. Middle right: NPDR case. Right: PDR case. The nailfold capillaroscopy parameters show the tendency towards decreasing, shortening, narrowing, and turbidity with progression from no DR to PDR

Representative cases of control subjects and patients with DR are shown in Fig. 1. The NC parameters varied depending on the presence or absence of diabetes and also on the severity of DR.

**Fig. 2:**
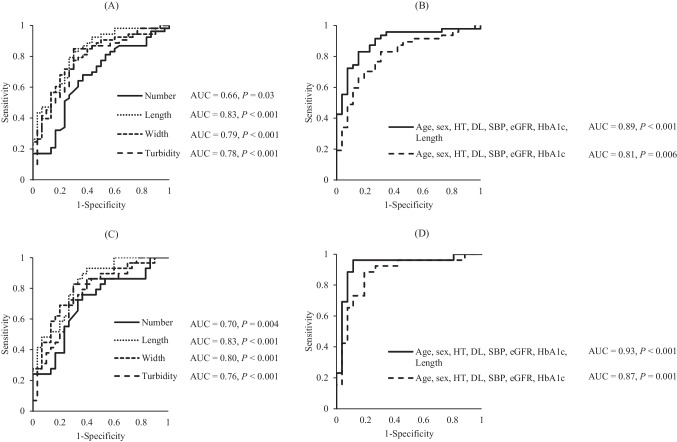
ROC curves to discriminate DR and PDR, (**A**) ROC curve comparing the AUC for each nailfold capillaroscopy parameter to discriminate DR. The AUC was highest for NC length (AUC = 0.83) among all NC parameters. (**B**) ROC curve comparing the AUC for systemic characteristics (age, sex, HT, DL, SBP, eGFR, and HbA1c) and systemic characteristics combined with NC length to discriminate DR. The AUC was higher for the systemic characteristics combined with NC length (AUC = 0.89) than for the systemic characteristics alone (AUC = 0.81). The discriminatory power for DR was thus significantly improved (*P* = 0.03) by adding NC length to the systemic findings. (**C**) ROC curve comparing the AUC for each nailfold capillaroscopy parameter to discriminate PDR. The AUC was highest for NC length (AUC = 0.83) among all NC parameters. (**D**) ROC curve comparing the AUC for the systemic characteristics and systemic characteristics combined with NC length to discriminate PDR. The AUCs were 0.87 for the systemic characteristics alone and 0.93 for the systemic characteristics combined with NC length

The results of a logistic regression analysis of NC parameters affecting the presence of DR and PDR in diabetic patients are shown in Table 3. The analysis showed that shortening of the NCs, narrowing of the NCs, and the NCs being turbid were each significantly related to the presence of DR (Table 3). Furthermore, the analysis showed that a decrease in the number of NCs, shortening of the NCs, narrowing of the NCs, and the NCs being turbid were each significantly related to the presence of PDR (Table 4). The ROC curves, based on the result of logistic regression, for DR and PDR with NC parameters alone, and the ROC curves when NC length was added to systemic findings, are shown in Fig. 2. For DR, the AUC of each NC parameter was as follows: 0.66 (95% CI 0.53‐0.77, *P* = 0.03) for number, 0.83 (95% CI 0.71‐0.90, *P* < 0.001) for length, 0.79 (95% CI 0.67‐0.87, *P* < 0.001) for width, and 0.78 (95% CI 0.66‐0.87, *P* < 0.001) for turbidity (Fig. 2A). When adding length to systemic risk factors, which included age, sex, HT, DL, SBP, eGFR, and HbA1c, the AUC increased from 0.81 (95% CI 0.68‐0.89, *P* = 0.006) to 0.89 (95% CI 0.79‐0.95, *P* < 0.001). This difference was statistically significant (*P* = 0.03) (Fig. 2B). For PDR, the AUC of each NC parameter was as follows: 0.70 (95% CI 0.55‐0.82, *P* = 0.004) for number, 0.83 (95% CI 0.70-0.91, *P* < 0.001) for length, 0.80 (95% CI 0.66-0.89, *P* < 0.001) for width, and 0.76 (95% CI 0.62-0.86, *P* < 0.001) for turbidity (Fig. 2C). When adding length to the systemic risk factors, the AUC increased from 0.87 (95% CI 0.73-0.95, *P* = 0.001) to 0.93 (95% CI 0.79-0.98, *P* < 0.001) (no significant difference) (Fig. 2D).

**Table 3:** Logistic regression analysis of independent variables affecting the presence of DR in diabetic patients

Variables			
Dependent	Independent	Adjusted OR (95% CI)	*P* value
DR	NC parameters		
	Number	0.58 (0.32-1.06)	0.08
	Length	0.24 (0.11-0.55)	< 0.001
	Width	0.26 (0.11-0.60)	0.002
	Turbidity	0.40 (0.21-0.75)	0.005

*OR*, odds ratio; *CI*, confidence interval; *DR*, diabetic retinopathy; *NC*, nailfold capillary. Differences were considered significant at the *P* < 0.05 level. All NC parameters were adjusted by age, sex, HT, DL, SBP, eGFR, and HbA_1c_

**Table 4:** Logistic regression analysis of independent variables affecting the presence of PDR in diabetic patients

Variables			
Dependent	Independent	Adjusted OR (95% CI)	*P* value
PDR	NC parameters		
	Number	0.26 (0.090-0.77)	0.02
	Length	0.17 (0.047-0.59)	0.005
	Width	0.26 (0.080-0.82)	0.02
	Turbidity	0.30 (0.11-0.79)	0.02

*OR*, odds ratio; *CI*, confidence interval; *PDR,* proliferative diabetic retinopathy; *NC,* nailfold capillary. Differences were considered significant at the *P* < 0.05 level. All NC parameters were adjusted by age, sex, HT, DL, SBP, eGFR, and HbA_1c_

## Discussion

The present study set out to determine whether noninvasive measurements of the NCs were associated with the presence and severity of DR in patients with type 2 diabetes. Our NC microscope device, the Kekkan-Bijin SC-10, could obtain NC images non-invasively and provide various NC parameters. We found that all four NC parameters were significantly lower in the diabetic patients than in the controls. There was a statistically significant difference in the NC parameters with the increasing severity of DR. Logistic regression analysis revealed that the systemic characteristics of age, sex, SBP, eGFR, HbA1c, and history of HT and DL could indicate the presence of DR (AUC = 0.81) and PDR (AUC = 0.87), while NC length could itself indicate the presence of DR and PDR (both AUCs = 0.83). Furthermore, the discriminative performance of DR was significantly improved by adding results for NC length to the systemic findings (AUC = 0.89).

### Underlying mechanism of NC and retinal capillary alterations

The underlying mechanism of NC alterations in diabetic patients remains unclear. Earlier studies have found that the pathogenesis of DR is driven by impaired function of vascular endothelial cells and loss of pericytes [12–18]. Pericytes are important in maintaining capillary stability, and their destabilization owing to chronic hyperglycemia or inflammation results in increased vascular permeability, capillary occlusion, and the emergence of microaneurysms in DR [13]. Additionally, damage to vascular endothelial cells may be linked to abnormal secretion and receptor kinetics of nitric oxide (NO). Consequently, NO, being a vasodilator, might have a reduced effect in the presence of vascular endothelial damage, thereby leading to vascular constriction [19, 20]. In the current study, a multivariate logistic analysis showed that the presence of DR and PDR were both closely associated with NC parameters, particularly NC length. Subsequently, NC length had an additive effect on systemic characteristics that indicated the presence of DR. Therefore, similar microvascular damage may occur in both NCs and retinal capillaries, and simple NC measurement without the need for a professional ophthalmological examination could be a screening tool to detect DR. Thus, although it remains unclear whether NC alterations precede decreased retinal microvascular changes in eyes with diabetes, the results of this study can be interpreted as implying that there is a shared pathological process that causes both NC alterations and DR.

Systemic oxidative stress may be involved in the onset of DR and may promote the progression of DR. Since oxidative stress has been linked to accelerated apoptosis of retinal capillary cells [21], it may be reasonable to consider that oxidative stress also influences changes in the NC parameters in DR patients. A previous study revealed that in addition to oxidative stress, increased serum levels of endothelin, a vasoconstricting peptide [22], and single nucleotide polymorphisms (SNPs) of caveolin 1, an inhibitor of tissue fibrosis [23], are associated with NC abnormalities in systemic sclerosis [24]. Advanced glycation end products (AGEs), an oxidative stress marker, were also reported to be significantly higher in diabetic patients than controls and to be closely associated with the increasing severity of DR [25]. Furthermore, the silent information regulator 1 (SIRT1) gene was confirmed to regulate AGE-induced pro-inflammatory cytokines and chemokines [26] and to be an important modulator of stress-induced vascular remodeling [27, 28]. Recently, it has been shown that SIRT1 negatively regulates angiogenesis, leading to delayed blood flow recovery after ischemia [29]. Thus, ischemia-induced oxidative stress in diabetic patients may trigger various molecular pathways of angiogenesis and capillary remodeling, causing morphological changes in the NCs and retinal capillaries.

### NC measurements to detect and evaluate DR

Several studies have demonstrated the relationships between NC alterations and DR. However, until now, most reports have evaluated these relationships qualitatively, e.g., a previous study of 108 individuals diagnosed with type 2 diabetes showed that a greater prevalence of intersecting NCs was correlated with a heightened risk of DR [7]. Another study of 216 patients with type 2 diabetes showed that NC alterations were significantly more common in these patients and that the tortuosity of the NCs was related to the presence of DR in a multivariate analysis [8]. Subsequently, an analysis of the relationship between our NC parameters and specific retinal microangiopathies, such as microaneurysms and dot hemorrhages, would also help explain the underlying mechanisms of NC and retinal capillary alterations. While the previous studies gave us novel insights into the association between NC parameters and DR, the relationship of NC alterations with the presence of DR remained unclear, because of the lack of a quantitative analysis of NC status. Unlike the past studies, our study evaluated NC alterations quantitatively using the nailfold capillaroscopy, Kekkan-Bijin SC-10. Thus, the current study, based on quantitative nailfold capillaroscopy, showed that NC parameters in diabetic patients were closely associated with the presence of DR and PDR in multivariate analyses. A previous study used another (video-based) method for nailfold capillaroscopy to quantitatively evaluate the NCs, finding that vasodilation of the NCs was a good indicator of DR; it could identify DR with an AUC of 0.75 [9]. However, this result contradicts ours. Several reasons for this are possible, including differences in the number of cases. The current study included 29 PDR patients (29/83: 34.9%), while the past study included only 4 PDR patients (4/62: 6.5%), which may have influenced the results. Another reason is that the past study did not consider renal function. As noted earlier, NO is a vasodilator whose production is reduced by impaired renal function [30]. In our study, we found that renal function decreased with the severity of DR. Therefore, it is reasonable to consider the possibility that NO production decreased as renal function worsened and the NCs narrowed. Furthermore, we were able to significantly improve the discriminatory power for DR by incorporating an NC parameter into conventional systemic risk factors, yielding an AUC of 0.89.

### Approaches other than NC measurements to detect and evaluate DR

In general, it is important to carefully control conventional risk factors, such as elevated HbA1c, HT, DL, and renal dysfunction, to prevent the development of DR. However, none of these systemic factors has a high discriminatory power for DR [31–34], and prior research suggests that HbA1c alone is an inadequate indicator of the onset of DR and its progression to PDR [25, 35, 36]. The current study also confirmed the inability to use HbA1c by itself for detecting DR, contrary to the excellent discriminatory ability of NC parameters. HbA1c represents blood glucose status over the past 2-3 months, indicating only the short-term effects of blood glucose control, while NC alterations represent the long-term accumulation of microvascular changes related to diabetes. As a result, the NC alterations may offer a more accurate representation of DR and PDR. Increased intima-media thickness (IMT) was reported to be closely associated with ocular ischemia in patients with type 2 diabetes, andbut the AUC of IMT to discriminate mild NPDR was not high at 0.67 [37]. The discriminative ability for DR and PDR of skin autofluorescence (SAF), which is easily measured with an AGE reader (DiagnOptics BV, Groningen, Netherlands) and reflects the long-term accumulation of AGEs, has reported to be high, at 0.76 for DR and 0.81 for PDR [25]. However, the additive effect on systemic findings to improve the discriminatory power has not yet been confirmed [25]. Furthermore, although optical coherence tomography angiography (OCTA) may be a promising tool for early DR diagnosis [38, 39], it is generally difficult to perform OCTA examinations accurately in cases of DR with DME, vitreous hemorrhage, and tractional retinal detachment. Thus, measurement of the NCs, which provides various parameters to directly assess the peripheral microvascular structure, could be a promising complementary method for detecting DR.

## Limitations

The current study had several limitations. First, because of the cross-sectional study design, it was not possible to discuss the time course of the association between NC alterations and DR. Second, previous reports have discussed the relationship between DR and other NC structural changes, such as avascular zones and hemorrhages [8, 9], but we did not examine these in the current study; we focused only on quantitative NC parameters. Third, we could not obtain information on the duration of diabetes or a history of smoking, which may be possible factors involved in the progression of DR. Fourth, total cholesterol and LDL were, paradoxically, lower in diabetic patients than in the normal controls, which may be explained by the fact that 41 patients (41/83: 49.4%) were taking oral antihyperlipidemic medication. Additionally, the ratio of glaucoma patients was significantly higher in the diabetes group than in the control group, although there were no differences in the ratio at each DR stage. We cannot rule out the possibility that this may have had some influence on the NC results. Finally, our sample size was relatively small and included only Asian subjects, and this may limit the generalizability of our findings to other geographic regions and ethnic groups. Therefore, validation using external samples is needed for establishing this prognostic model.

## Conclusion

The present study shows that nailfold capillaroscopy may be useful as a simple, non-invasive technique for evaluating the risk of DR and its severity. Our findings indicate that alterations in NC structure, such as capillary shortening, may be closely correlated with the presence of DR and PDR. NC measurements may have the potential to help screen for DR. Further research into the precise relationship and temporal associations between NC alterations and DR is necessary to fully understand our observations.

## Data Availability

The first author, Tatsu Okabe, had full access to all the data in the study. The data that support the findings of this study are available from the corresponding author, Hiroshi Kunikata, and the first investigator, Tatsu Okabe, upon reasonable request.
